# Large Recurrent Mediastinal Abscess Presenting With Cardiopulmonary Collapse

**DOI:** 10.7759/cureus.14653

**Published:** 2021-04-23

**Authors:** Brandon Kalivoda, Alexandra Lackey, Aashish Mainali, Jian Guan

**Affiliations:** 1 Internal Medicine, AdventHealth Orlando, Orlando, USA

**Keywords:** mediastinal abscess, mediastinal cyst

## Abstract

Mediastinal abscesses are rarely encountered but pose a potential threat for cardiopulmonary collapse given the close proximity of vital structures. Our focus is to illustrate a case of a mediastinal abscess that was promptly diagnosed and treated, leading to complete resolution of the airway and circulatory compromise. The proposed pathogeneses behind mediastinal abscesses are discussed at length.

## Introduction

Mediastinal infections can be a life-threatening and rare event encountered, especially when they are caused by a dental abscess or a foreign body that has perforated the esophagus. Mediastinitis is associated with a high mortality rate if the diagnosis is not quickly established and sufficient therapy is not provided [1]. Mediastinal masses encompass several clinical challenges due to the broad differential and potential for life-threatening complications. Housing the heart, esophagus, trachea, and major vessels, even slight interruptions of this cavity can have devastating consequences. We present a case of a 29-year-old female who presented to the emergency room with sudden-onset respiratory distress progressing rapidly to cardiopulmonary collapse secondary to a mediastinal abscess.

## Case presentation

A 29-year-old female presented to the emergency room with sudden onset of shortness of breath and cough productive of red sputum for three days. She offered no further complaints at the time of presentation and denied the use of alcohol and recreational drugs.

Her medical history was significant for a dental abscess that was treated 10 years ago. Additionally, approximately eight years ago, she had an episode of respiratory distress and was found to have a mediastinal abscess that was drained during a mediastinoscopy with complete resolution of symptoms. She denied any family history of malignancy.

Initial vital signs revealed a heart rate of 104, temperature of 99.4 °F, respiratory rate of 18, O_2_ saturation of 98% on room air, and blood pressure of 111/75. Physical exam revealed a well-developed and well-nourished female in mild respiratory distress with good oral hygiene without overt signs of an oral infection. Lungs were clear to auscultation bilaterally and cardiac examination revealed a regular heart rate and rhythm without murmurs, rubs, or gallops.

Laboratory workup revealed a mild leukocytosis with a white count of 11.08K, but the remainder of the results of the complete blood count with differentials, as well as a comprehensive metabolic panel, were within normal limits. 

**Figure 1:**
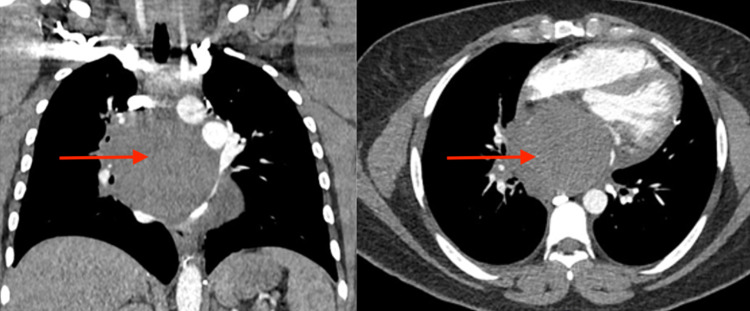
Coronal (left) and transverse (right) views of the posterior mediastinal mass (red arrows) prior to mediastinoscopy Mass dimensions: 7.8 cm x 8.8 cm x 7.8 cm

**Figure 2:**
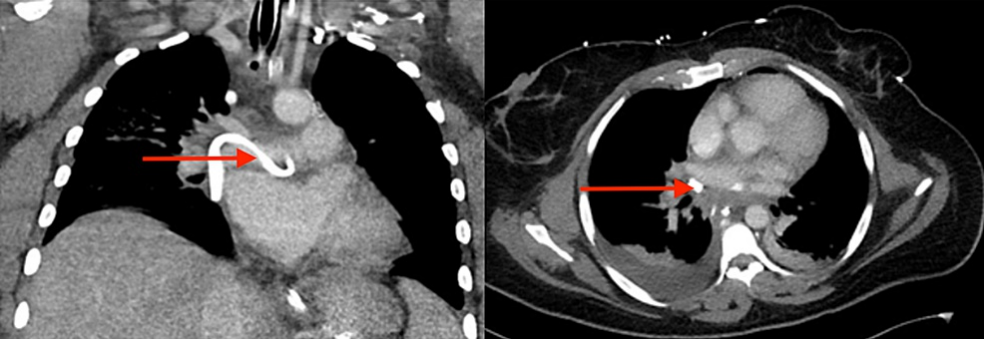
Coronal (left) and transverse (right) views of the posterior mediastinal mass after mediastinoscopy with drain placement (red arrows)

The patient was admitted for further evaluation of respiratory distress and progressively decompensated. Arterial blood gas was obtained 24 hours later, which showed pH 7.1, partial pressure of carbon dioxide (pCO2) 77, and partial pressure of oxygen (pO2) 100 on a non-rebreather. She was subsequently intubated for acute respiratory acidosis and transferred to the intensive care unit (ICU). She required maximal sedation with midazolam, propofol, and fentanyl, as well as paralysis with cisatracurium due to ventilator dyssynchrony. A chest CT angiography showed a large mediastinal mass centered in the subcarinal region with compression and effacement of multiple structures, most prominently the superior vena cava, left atrium, and pulmonary veins (Figure 1). There was also patchy consolidation in the right upper and middle lobes suspicious of infection. She was subsequently started on broad-spectrum antibiotics. Transthoracic echocardiogram showed extrinsic compression of the left atrium from the mediastinal mass with an ejection fraction of 55%-60% and right ventricular systolic pressure of 36 mmHg. She underwent endobronchial ultrasound (EBUS) with fine-needle aspiration (FNA). Cultures grew coagulase-negative Staphylococcus, raising concern for possible abscess formation. Mediastinoscopy was performed and revealed a cystic lesion filled with purulent material. Approximately 150 mL of fluid was removed and a drain was placed (Figure 2). Cultures of the purulent fluid grew Prevotella species and the antibiotic regimen was deescalated to linezolid and ampicillin/sulbactam. Four days following mediastinoscopy, the patient was successfully extubated. Her respiratory and cardiac status continued to improve, and she was discharged home with ongoing antibiotic therapy (amoxicillin/clavulanic acid, metronidazole, and linezolid).

## Discussion

Mediastinal masses encompass many different etiologies. Their presentations are broad, ranging from asymptomatic found incidentally on imaging to life-threatening cardiorespiratory compromise as in our patient’s case. The differential diagnoses and subsequent workup can be formulated based on the location of the mass within the mediastinum. Anterior masses encompass the “four Ts”: thymoma, teratoma, thyroid goiter or neoplasm, and “terrible” lymphoma. Posterior masses, like the one presented in our case, may result from a neurogenic tumor, hiatal hernia, aortic aneurysm, embryologic cyst, or abscess.

It is important to note that mediastinal masses pose a direct threat to the patient’s airway. Intubation of these patients should be carefully planned with an anesthesiologist. Sedation and paralysis of these patients can result in life-threatening airway collapse due to the elimination of the transpleural pressure gradient as well as smooth muscle relaxation, which allows for increased compressibility of vital structures by the mass. It is, therefore, reasonable to consider techniques including awake fiberoptic intubation. If time permits, CT imaging and pulmonary function tests can help guide airway management [2].

Given this patient’s hemodynamic and respiratory instability, common and rare etiologies of her mass were promptly and carefully considered. Pulmonology was immediately involved in this case so that tissue diagnosis could be achieved through EBUS. Additionally, because of the potential for malignancy and the possibly emergent need for mass debulking or radiotherapy, oncology was consulted early in our patient’s care. Without a prompt multi-disciplinary approach, the patient would have undoubtedly suffered progressive hemodynamic and respiratory demise.

Initial surgical cultures from the FNA collected during EBUS grew coagulase-negative Staphylococci (CoNS), a type of staph commonly found as part of the normal flora of the skin. The other organism that was identified during mediastinoscopy was a Prevotella species, a genus of bacteria involving obligate, anaerobic, gram-negative bacteria that are commensal organisms of the oral cavity, gut, and vaginal mucosa [3].

This case brings into question the possible origin of this patient’s infection. Mediastinal abscesses are usually the result of esophageal perforation, cardiac surgery, or pneumonia with local spread into the mediastinum, none of which seemed to apply to our patient.

Occasionally, mediastinitis and abscess formation may result from caudal extension of a retropharyngeal or oral infection [4]. Anaerobic, gram-negative organisms, such as Prevotella, as well as Fusobacterium, Parvimonas, and Streptococcus, are associated with oral infections [5]. Dental abscesses harboring these anaerobes can dissect into the posterior mediastinum through fascial planes and the prevertebral space [6]. This explanation seems to be the most reasonable explanation for the presence of Prevotella given her history of a dental infection preceding the first mediastinoscopy.

Additionally, endobronchial ultrasound with transbronchial needle aspiration is recognized as a means of introducing infection into the mediastinum [7]. It is unclear whether the CoNS was inoculated into the mediastinum during the prior mediastinoscopy or was a contamination during the most recent EBUS with FNA.

Another consideration in this patient’s case is the possibility of a congenital mediastinal cyst that eventually became a host for infection. Embryologic origins behind mediastinal cysts include a bronchogenic cyst, intramural esophageal cyst, and enteric cyst. Cystic lesions of the mediastinum constitute 10%-15% of all radiographically detected mediastinal masses [8].

The standard approach to the treatment of a mediastinal abscess is surgical drainage. Katayama et al. describe a similar case of a mediastinal abscess that was treated with surgical extirpation and ethanol instillation. They describe this method as the treatment of choice for such lesions [4]. Another case described in the literature involves mediastinitis caused by an odontogenic abscess with Prevotella corporis. This patient was treated successfully with surgical drainage, antibiotics, and subsequent hyperbaric oxygen therapy. Aggressive treatment is needed because the mortality rate of a cervical abscess secondary to odontogenic infection and complicated by mediastinitis varies from 10% to 40% [9].

## Conclusions

In conclusion, mediastinal abscesses should be diagnosed promptly in order to initiate the appropriate therapy. Without surgical intervention, the patient would not have survived due to cardiopulmonary collapse. After thorough history-taking, it was elicited that she had a history of a dental infection likely leading to the first and subsequent episodes of mediastinal abscesses.
